# Salivary Metabolomics in the Diagnosis and Monitoring of Neurodegenerative Dementia

**DOI:** 10.3390/metabo13020233

**Published:** 2023-02-04

**Authors:** Eelis Hyvärinen, Eino Solje, Jouko Vepsäläinen, Arja Kullaa, Tuulia Tynkkynen

**Affiliations:** 1 Institute of Dentistry, University of Eastern Finland, 70210 Kuopio, Finland; 2 Institute of Clinical Medicine, Neurology, University of Eastern Finland, 70210 Kuopio, Finland; 3 Neuro Center, Neurology, Kuopio University Hospital, 70210 Kuopio, Finland; 4 NMR Metabolomics Laboratory, School of Pharmacy, University of Eastern Finland, 70210 Kuopio, Finland

**Keywords:** neurodegenerative diseases, dementia, saliva, metabolites, NMR spectroscopy, mass spectrometry

## Abstract

Millions of people suffer with dementia worldwide. However, early diagnosis of neurodegenerative diseases/dementia (NDD) is difficult, and no specific biomarkers have been found. This study aims to review the applications of salivary metabolomics in diagnostics and the treatment monitoring of NDD A literature search of suitable studies was executed so that a total of 29 original research articles were included in the present review. Spectroscopic methods, mainly nuclear magnetic resonance (NMR) spectroscopy and mass spectrometry, give us a broad view of changes in salivary metabolites in neurodegenerative diseases. The role of different salivary metabolites in brain function is discussed. Further studies with larger patient cohorts should be carried out to investigate the association between salivary metabolites and brain function and thus learn more about the complicated pathways in the human body.

## 1. Introduction

Approximately 55 million people suffer with dementia worldwide. Dementia is a syndrome affecting memory, thinking, orientation, comprehension, calculation, learning capacity, language and judgement [1]. Most commonly, dementia is caused by progressive diseases inducing neurodegeneration including Alzheimer’s disease (AD), frontotemporal dementia (FTD), vascular dementia (VaD) and alpha synucleinopathies: dementia with Lewy bodies (DLB) and Parkinson’s disease dementia (PDD). AD accounts for about 70% of all dementia cases, and the number of patients suffering from dementia is increasing due to increasing average lifetime [2]. Many research results suggest that pathophysiological changes initiate at least 10 to 25 years before the onset of dementia symptoms [3]. 

Diagnosis of neurodegenerative diseases is difficult, especially in the pre-clinical stages [4,5]. Many biomarkers based on imaging and cerebrospinal fluid (CSF) have been suggested to be positively associated with early diagnosis, but disease specificity is lacking [6]. In cognitively asymptomatic individuals with positive biomarkers for AD, the lifetime dementia risk is estimated to be from 5% to 42% [7]. Blood neurofilament light chain (NfL) is suggested to be a biomarker for neurodegenerative disorders, but it is not disease-specific and rather reflects neuronal damage in general [8]. Hence, there is an urgent need for new diagnostic, prognostic and monitoring biomarker innovations.

Saliva, a complex biofluid with a high variety of molecules, mainly consists of water (99%) and inorganic and organic substances [9]. Saliva is secreted from three pairs of major salivary glands (i.e., parotid, submandibular, sublingual) and numerous minor salivary glands throughout the oral cavity and pharynx. The functions of salivary glands are controlled by the sympathetic/parasympathetic nervous system. Primary saliva is produced from blood components by the acinar cells via transcellular diffusion and via the tight cell junctions of these cells [10]. Before entering the mouth, saliva is modified by the ductal cells, including the intercalated, striated and excretory cells, via reabsorption to the bloodstream. Furthermore, saliva flow rate, oral microbiota, oral mucosal transudate, immune cells and other environmental factors have an impact on the final composition of whole mouth saliva [10,11,12]. Saliva contains several compounds that are involved in oral health maintenance. In addition to oral diseases, the origin of saliva enables salivary diagnostics of systemic diseases [13].

**Figure 1:**
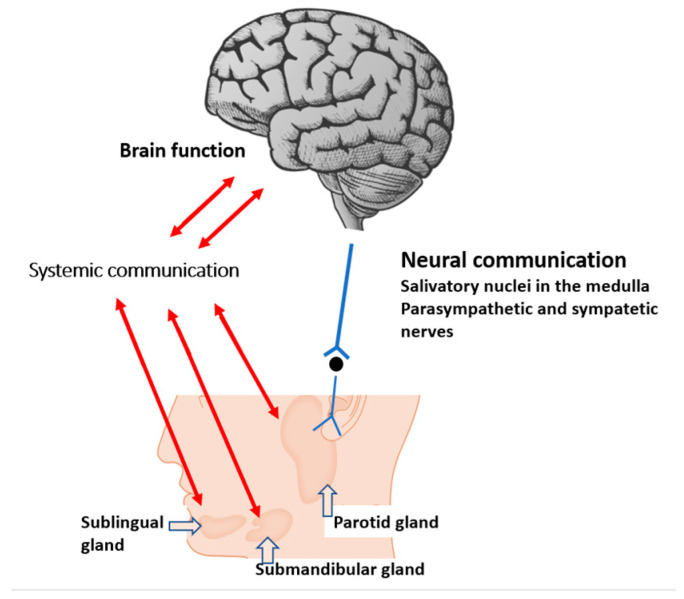
Systemic and neural pathways linking the salivary gland with brain function. Metabolites play a central role in systemic communication.

Salivary glands work as an exocrine (external secretions as saliva) and endocrine organ. Some of the salivary products are transferred into the bloodstream via endocrine mechanisms and communicate with other organs, including the brain (Figure 1) [14]. Hence, saliva is an accessible source of information as a ‘mirror of the body’ and a promising biofluid for the diagnosis and monitoring of human diseases because of its bidirectional mechanisms. Furthermore, in contrast to blood or CSF, the collection of saliva is non-invasive and safe.

Salivary analysis requires precise methods due to the low concentration of salivary components. Metabolites provide comprehensive information about the cellular functions of oral tissues and changes in the phenotype of cells or tissues in response to genetic or environmental changes. The most common methods are enzyme-linked immunosorbent assays (ELISA) and different spectroscopic methods. Mass spectrometry (MS) and nuclear magnetic resonance (NMR) spectroscopy are frequently used methods in saliva research [13]. Mass spectrometry is commonly used in conjunction with either two-dimensional gas chromatography (2DGC-MS) or high-performance liquid chromatography (HPLC-MS) [13]. NMR spectroscopy is based on the behaviour of magnetically active atomic nuclei, e.g., ^1^H or ^13^C, in an external magnetic field. Identification of small molecules is possible because most compounds have highly characteristic resonance frequencies [15]. Additionally, Raman spectroscopy, Fourier-transform infrared (FTIR) spectroscopy and photoacoustic spectroscopy (PAS) have been used in salivary research [16,17].

Because of the precise molecular identification, spectroscopic methods are potential diagnostic tools in the field of salivary metabolomics. This study aimed to conduct this literature review on the applications of salivary metabolites in diagnostics and treatment monitoring of neurodegenerative diseases in order to form a basis for further studies.

## 2. Materials and Methods

We divided different neurodegenerative diseases into four groups: AD, FTD, VaD and alpha synucleinopathies, i.e., DLB and PDD.

### Search Strategy and Study Selection

A literature search of suitable studies was conducted using the PubMed and Web of Science databases, utilizing the following keywords: ”Alzheimer’s disease” AND “saliva*” AND (“biomarker*” OR “metabolite*”); “dementia” AND “Lewy bod*” AND “saliva*” AND (“biomarker*” OR “metabolite*”); (“frontotemporal dementia” OR “frontotemporal lobe degeneration”) AND “saliva*” AND (“biomarker*” OR “metabolite*”); “vascular dementia” AND “saliva*” AND (“biomarker*” OR “metabolite*”); “Parkinson’s” AND “dementia” AND “saliva*” AND (“biomarker*” OR “metabolite*”). The search was performed without the limitations of publication year. The searches were conducted in December 2021. Additional searches (n = 5) were conducted until October 2022.

The literature search was executed in two phases. First, the following validity criteria were used for screening the titles of the articles: only English; publication year 2000 or later; saliva must be examined in the wanted disease. In the second phase, screening abstracts of the articles, literature reviews, conference abstracts and articles about non-human material were excluded. 

**Figure 2:**
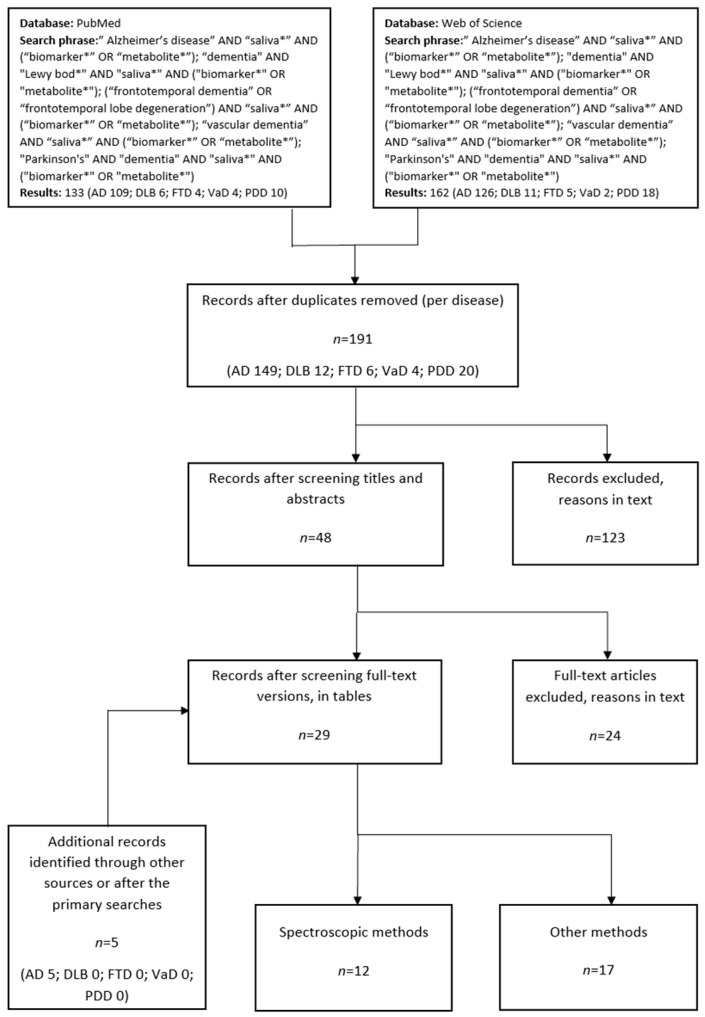
Flow chart of the English literature review process in the time range January 2000–December 2021. Additional records were identified until October 2022. (AD = Alzheimer disease; DLB = dementia with Lewy bodies; FTD = frontotemporal dementia; VaD = vascular dementia; PDD = Parkinson’s disease dementia). * mark means a cut-off mark commonly used in a literary search that allows complete search of the subject in question.

Two authors (E.H. and A.K.) independently appraised full-text versions of the selected articles and then together excluded studies that did not handle the content of the present review. We excluded articles that handled only methodological issues or did not contain any metabolomic results. The reference lists of selected articles were manually reviewed to find suitable studies outside the literature review (Figure 2). 

## 3. Results

A total of 29 original research articles were included in the present review, of which 12 addressed spectroscopic methods (Figure 2).

Different methods, mostly ELISA, have been used to study salivary biomarkers (Table 1). These methods often concentrate on single biomarkers that have earlier been associated with the diseases, such as amyloid-β42 (Aβ), t-tau and lactoferrin in AD. In plasma and CSF, NfL has been shown to be a promising biomarker for neurodegeneration. However, a similar trend has not been found using saliva samples [18].

Studies with MS and NMR spectroscopy involve many salivary metabolites associated with different stages of neurodegenerative diseases. In Table 2, we present all metabolites that have been shown to be related to neurodegenerative diseases. 

Most spectroscopic studies investigate MCI and AD. We found just one article that addressed vascular dementia [36] and another that addressed FTD and dementia with Lewy bodies [37]. Two articles investigated Parkinson’s disease using spectroscopic methods [38,39]. These two articles are excluded because they handled only Parkinson’s disease and did not differentiate patients with cognitive symptoms (PDD). To our knowledge, there are no studies of Parkinson’s disease dementia using saliva samples and spectroscopic methods.

**Table 2:** Changes in salivary metabolites when comparing mild cognitive impairment (MCI) with healthy controls (HC), Alzheimer’s disease (AD) with HC, and AD with MCI according to previous studies using spectroscopic methods (NMR and MS). Two studies compared multiple neurodegenerative dementia (NDD) with HC (36,37).

Disease (N)	Method	Metabolites (Elevated/Lowered)	Reference
MCI (8) vs. HC (12)	NMR	acetone, imidazole galactose	[40]
MCI (20) vs. HC (20)	LC-FTICR-MS	taurine	[41]
MCI (25) vs. HC (25)	FIA-MS/MS	acyl-alkyl phosphatidylcholines	[42]
MCI (20) vs. HC (40)	GC-MS	hydroxyphenyl lactate, tyramine, tyrosol cholesterol	[43]
MCI (21) vs. HC (19)	LC-MS/MS	transthyretin	[44]
MCI (59) vs. HC (131)	MALDI-TOF/TOF MS	lactoferrin	[45]
MCI (20)/AD (20) vs. HC (40)	GC-MS	rhamnose, L-tyrosine, L-fucose, L-ornithine, L-aspartate, serotonin	[43]
AD (9) vs. HC (12)	NMR	acetone, propionate	[40]
AD (116) vs. HC (131)	MALDI-TOF/TOF MS	lactoferrin	[45]
AD (21) vs. HC (38)	MALDI-TOF- MS/MS	p-tau/t-tau ratio	[46]
AD (29) vs. HC (45)	LC-MS	phenylalanyl-proline, phenylalanyl-phenylalanine, tryptophyl-tyrosine, urocanic acid	[47]
AD (256) vs. HC (218)	FUPLC-MS	ornithine, phenyllactic acid, sphinganine-1-phosphate 3-dehydrocarnitine, hypoxanthine, inosine	[48]
AD (20) vs. HC (40)	GC-MS	aspartate, ornithine, phenylalanine, pyruvate, tyrosine, putrescine, cholesterol citrate, fumarate, succinate	[43]
AD (17) vs. HC (19)	LC-MS/MS	transthyretin	[44]
AD (25) vs. HC (25)	FIA-MS/MS	acyl-alkyl phosphatidylcholines	[42]
AD (9) vs. MCI (8)	NMR	5-aminopentanoate, creatine	[40]
AD (29) vs. MCI (35)	LC-MS	alanyl-phenylalanine, phenylalanyl-glycine, phenylalanyl-proline	[47]
AD (660) vs. MCI (583)	FUPLC-MS	cytidine, L-glutamate, ornithine, phenyllactic acid, pyroglutamate, L-tryptophan, sphinganine-1-phosphate 3-dehydrocarnitine, hypoxanthine, inosine	[49]
Dementia (17) (13 AD + 4 VaD) vs. HC (34)	NMR	acetic acid, histamine, propionate dimethyl sulfone, glycerol, succinate, taurine	[36]
Dementia (10) (3 AD + 4 FTD + 3 DLB) vs. HC (9)	CE-TOF-MS	arginine, tyrosine	[37]

N = number of subjects; AD = Alzheimer’s disease; MCI = mild cognitive impairment; VaD = vascular dementia; FTD = frontotemporal dementia; DLB = dementia with Lewy bodies; HC = healthy controls; LC = liquid chromatography; FTICR = Fourier transform ion cyclotron resonance; MS = mass spectrometry; FIA = flow injection analysis; MS/MS = tandem mass spectrometry; GC = gas chromatography; MALDI = matrix-assisted laser desorption/ionization; TOF = time-of-flight; FUPLC = faster ultra-performance liquid chromatography; CE = capillary electrophoresis; TOF/TOF = tandem time-of-flight; NMR = nuclear magnetic resonance spectroscopy; t-tau = total tau; p-tau = phosphorylated tau.

## 4. Discussion

In this review, we analysed the literature on the association between neurodegenerative dementia and salivary metabolites. We divided neurodegenerative diseases leading to dementia into different types: AD, FTD, VaD and alpha synucleinopathies: DLB and PDD. Most of the articles discussed AD and MCI. Only one study analysed AD and VaD [36] and only one analysed AD and FTD [37]. Only two articles [38,39] handled PD, but did not differentiate patients according to cognitive symptoms (PDD). In future studies, the underlying neuropathology or pathophysiologial process in the research subjects should be established using neuropathological analysis or modern beyond-state-of-the-art methods. In particular, CSF RT-quIC [8] in the identification of the underlying proteinopathy and transcranial magnetic stimulation [50] in the recognition of the disease-specific neurotransmitter system deficit could increase the validity of saliva biomarker studies.

Some single salivary metabolites, including Aβ, t-tau and lactoferrin, are associated with AD (Table 1). Increased salivary Aβ is shown in AD patients, but is not evident in studies with MS and NMR spectroscopy. Decreased salivary lactoferrin and increased t-tau are shown also with MS in some studies [45,46]. Lactoferrin, one component of the innate defence mechanism of saliva, is produced via salivary glands and also from gingival cervicular fluid, and it is active against oral microbes [10]. Hence, it can be a biomarker of gingivitis and periodontitis. 

With spectroscopic methods, we can obtain a wide scale of different salivary metabolites and thus identify disease-associated changes in oral metabolism as a mirror of whole human body physiology. François et al. [43] discovered that serotonin is increased in patients with AD versus MCI and healthy controls. Tryptophan is a precursor for serotonin [51], and L-tryptophan has been discovered to be elevated in AD versus MCI [49]. Serotonin affects nearly all human behavioural processes, but a major amount of serotonin is found outside the central nervous system. Approximately 95% of total body serotonin is produced by the intestinal enterochromaffin cells [52] and therefore it may not be a promising salivary biomarker for AD. In addition, high levels of tryptophan-tyrosine dipeptide in the saliva of AD patients might indicate memory impairments due to altered dopaminergic activity [53]. In the future, studies of serotonin, tryptophan and dipeptides in the saliva might indicate pathway changes and episodic memory impairment in patients with AD.

Studied with NMR spectroscopy, salivary propionate has been found to be upregulated in patients with AD when compared to controls [36,40]. However, propionate is also increased in inflammatory oral diseases, including periodontal diseases and dental caries, therefore its effectiveness as a specific salivary biomarker for neurodegenerative diseases is questionable. On the other hand, periodontitis and tooth loss have been shown to increase the risk of dementia [54,55,56]. Gut microbiota and their metabolites, like propionate, have been mentioned in mediating brain function [57]. Salivary propionate is produced by oral bacteria [12], but the link between salivary propionate and the brain has not been studied. In addition to inflammatory diseases, oral dysbiosis together with salivary metabolomics could be one target to study further in patients with neurodegenerative dementia.

Salivary metabolites mainly reflect the oral microbiome. Concentrations of some metabolites, including short chain fatty acids (SCFAs: acetate, butyrate, propionate, formate), correlate with salivary bacterial load [12]. On the other hand, SCFAs, as immune-regulatory metabolites, can stimulate the autonomic nervous system [58,59]. These metabolites, produced by proteolytic bacteria, are associated with periodontitis [60,61] and some of these metabolites have also been found in patients with MCI and VaD vs. controls [36,40]. In this regard, we hypothesize that salivary SCFAs circulate in the blood and can cause low-level systemic inflammation and associate with brain function. The biological mechanisms and systemic communication between the brain and oral health are yet unknown. Hence, the association between inflammatory oral diseases and brain function presents a target for further study on salivary metabolites. The role of salivary SCFAs in the mouth–brain axis needs more investigation.

The level of salivary taurine was lower in patients with MCI [41] and AD/VaD [36] when compared to controls. Taurine has numerous functions in the nervous system, including neurotransmission, neuromodulation and osmoregulation, and it prevents the neurotoxicity of Aβ [62].

Salivary histamine was increased in patients with AD and VaD versus controls [36]. The central histaminergic system in the brain plays a major role in basic body functions, such as the sleep-waking cycle and learning, and has been reported to be involved in AD [63]. In addition to histaminergic neurons, histamine is primarily produced by mast cells, basophils, and enterochromaffin-like cells in the stomach [64]. 

Figueira et al. [36] also conducted a follow-up study with 28 dementia patients (14 AD, 11 VaD, 3 DLB or FTD) and 60 controls. They managed to differentiate controls and healthy, pre-dementia patients using seven metabolites: acetic acid, histamine and propionate increased, whereas dimethyl sulfone, glycerol, succinate and taurine decreased (Table 2). Future studies should increasingly concentrate on these kinds of follow-up studies to determine the specific and sensitive biomarkers of early stages of the diseases.

Recent metabolomic studies have often been conducted with relatively small study populations. To verify these results, multi-centre investigations with larger cross-sectional populations are needed. Such projects would also enable longitudinal, long-term follow-up studies and include more background information on patient health. Furthermore, an important object of biomarker research in neurodegenerative dementia is to compare the validated metabolic biomarkers from multiple biofluids including blood, CSF and saliva. Standardized collection and storage methods and increasing interest in saliva research could make high-quality saliva research possible in the future. 

Salivary metabolites have recently been investigated with spectroscopic methods in different diseases [13]. However, the collection methods vary considerably. Stimulation of salivary secretion is necessary with some patients with hyposalivation, e.g., elderly people. Figueira et al. [65] highlighted in their study that comparable results are obtained only by using the same sample collection methods. We recommend collecting (masticatory or gustatory) stimulated saliva samples. Thus, the sample volume is higher on average and patients with lowered salivary secretion can be involved in the study.

The studies included in this review demonstrated the multifunctional character of salivary metabolites and their association with neurodegenerative dementia. MS and NMR spectroscopy provide more information about salivary metabolomic profiles and pathways in the oral cavity than analysis of simple metabolites. The biological mechanisms and systemic communication between the brain and oral health are yet unknown and need more studies in larger patient and multicentre cohorts. 

## 5. Conclusions

Spectroscopic methods (NMR, MS) give us a broad view of changes in salivary metabolites in neurodegenerative diseases and deepen our knowledge of the systemic communication between the oral cavity and the brain. Further studies with larger patient cohorts should be carried out to investigate the association between salivary metabolites and brain function and thus learn more about the complicated pathways in the human body. 

## Figures and Tables

**Table 1:** Some salivary metabolites that have been studied with various neurodegenerative diseases using different methods.

Disease	Metabolites (Elevated/No Association/Lowered)	Method
AD	amyloid-β42	ELISA [19,20,21,22]
AD	amyloid-β42	ELISA [23]
AD	amyloid-β42	Luminex assay [24]
AD	complement C4	Luminex assay [24]
AD	t-tau	ELISA [23] Lumipulse technology [25]
AD	p-tau/t-tau ratio	Antibodies + Western Blot analysis [26] ELISA [22]
AD	SIRT1, SIRT3, SIRT6	ELISA [27]
AD	glutathione	Colorimetric method [28]
AD	IgA	ELISA [29]
AD	cortisol	ELISA [29]
AD	cortisol	RIA [30]
AD, MCI	t-tau	Single molecule array [31]
AD, MCI	GFAP	ELISA [32] quantitative Dot Blot analysis [32] SDS-PAGE + Western Blot analysis [32]
AD, MCI	amyloid-β42	Magnetoimmunoassay [33]
AD, FTD	lactoferrin	ELISA [34]
AD, MCI, FTD, DLB, VaD, PDD	lactoferrin	ELISA [35]

AD = Alzheimer’s disease; MCI = mild cognitive impairment; FTD = frontotemporal dementia; DLB = dementia with Lewy bodies; VaD = vascular dementia; PDD = Parkinson’s disease dementia; HC = healthy controls; t-tau = total tau; p-tau = phosphorylated tau; SIRT = sirtuin; IgA = immunoglobulin A; GFAP = glial fibrillary acidic protein; ELISA = enzyme-linked immunosorbent assay; SDS-PAGE = SDS polyacrylamide gel electrophoresis; RIA = radioimmunoassay kit.
